# Continuous Glucose Monitoring and Physical Activity

**DOI:** 10.3390/ijerph191912296

**Published:** 2022-09-28

**Authors:** Oliver Schubert-Olesen, Jens Kröger, Thorsten Siegmund, Ulrike Thurm, Martin Halle

**Affiliations:** 1 Center for Diabetology Hamburg City, 20095 Hamburg, Germany; 2 Center of Digital Diabetology Hamburg, 21029 Hamburg, Germany; 3 Diabetes, Hormones and Metabolism Center, Private Practice at the Isar Clinic, 80331 Munich, Germany; 4 IDAA, Diabetic Athletes Association, 12621 Berlin, Germany; 5 Department of Preventive Sports Medicine and Sports Cardiology, University Hospital Klinikum Rechts der Isar, Technical University of Munich, 80992 Munich, Germany

**Keywords:** continuous glucose monitoring, physical activity, diabetes mellitus

## Abstract

Continuous glucose monitoring (CGM) use has several potential positive effects on diabetes management. These benefits are, e.g., increased time in range (TIR), optimized therapy, and developed documentation. Physical activity is a recommended intervention tool in diabetes management, especially for people with type 2 diabetes (T2D). The benefits of physical activity for people with diabetes can be seen as an improvement of glycemic control, glycemic variability, and the reduction of insulin resistance. In relation to the physical activity of people with T2D, the benefits of CGM use can even be increased, and CGM can be a helpful tool to prevent adverse events due to physical activity of people with diabetes, such as hypoglycemic events and nocturnal hypoglycemia after sports. This narrative review aims to provide solid recommendations for the use of CGM in everyday life physical activities based on the noted benefits and to give a general overview of the guidelines on physical activity and CGM use for people with diabetes.

## 1. Physical Activity and Diabetes

Physical activity is generally defined as any movement of the body driven by skeletal muscles under which energy expenditure takes place. Exercise is a physical activity that is structured, planned, and repeated with the aim of increasing physical fitness [1].

In diabetes management, especially for people with type 2 diabetes (T2D), physical activity is considered an intervention tool. Physical activity affects people’s general health and physical well-being. Physical inactivity and exercise deficiency syndrome (EDS), along with smoking, are the strongest predictors of mortality in general population [2,3]. It has been shown that one in ten deaths can be attributed to physical inactivity and its consequences, such as cardiovascular (CV) disease [2,3]. EDS, a lack of physical exercise, results in low cardiopulmonary fitness. This has a particularly detrimental effect on chronically ill people, such as those with diabetes [3].

The most frequently cited reasons for a lack of physical activity in everyday life in a survey of 1003 German citizens in 2014 were: distances considered as too long, e.g., to go shopping (53%), lack of time (46%), illness, physical limitations or obesity (26%), lack of motivation (23%), and a general aversion to physical activity (3%). In 2012, in a study by the Robert Koch Institute, only 20% of respondents achieved the minimum recommendation of 2.5 h of physical activity per week [4]. However, the proportion of physical activity has increased significantly over the last 10 years. A total of 57% of men and 48% of women reported being physically active for at least one hour a week [4]. On average, adults under 65 years of age moved between 5400–18,000 steps per day [5]. Younger adults between the ages of 20 and 50 years walked on average 7000–13,000 steps per day [5].

The consequence of this lack of exercise is often obesity. Approximately 25% of the German population was classified as obese in 2018. Obesity in particular is a risk factor for T2D. For every 1 kg/m^2^ higher body mass index (BMI), the risk of developing T2D is increased by about 20% [6]. In 2015, the number of people diagnosed with T2D in Germany was 6.9 million. Whereas, the total number of people with type 1 diabetes (T1D) was 166,000 (134,000 adults and 32,000 children) in Germany [7,8]. The projected increase in the number of people with T2D for the year 2040 is approximately 21%, which would correspond to a total number of 8.3 million German citizens [7].

Even moderate forms of physical activity can show positive long-term effects on glycemic control, represented as HbA1c [9,10]. Individual training sessions can also lead to short-term changes in glycemic values, which can be represented as time in range (TIR). These can be visualized for the exercising person with diabetes by using continuous glucose monitoring (CGM) systems and used for diabetes management by calculating the TIR [11]. CGM can also help to quickly detect or avoid short-term negative effects of training sessions such as hypoglycemia [11].

The motivation of people with diabetes to change their exercise behavior in everyday life in the long term can be achieved through motivational interviewing and the provision of practical tips. The resulting increase in self-efficacy can positively influence the experience of the disease and thus support the maintenance of healthy behaviors [12]. Here, fears of the negative consequences of physical activity should be reduced. Regular evaluation discussions of the measures taken regarding therapy adherence and intensity should take place during the course of treatment [12,13,14].

The aim of this narrative review is to present the potential influences of CGM measurements in relation to exercise in people with diabetes, to provide an overview of current recommendations on physical activity and CGM use for people with diabetes, and to highlight the benefits of CGM in relation to exercise [15].

## 2. Benefits and Effects of CGM in the Context of Physical Activity—Which Measurement Parameters Can Be Presented in an Optimized Way?

CGM measurement parameters can make even short-term changes in glycemic control visible. With CGM, measuring intervals every 5 min is possible also during sleeping phases. This enables us to give a more detailed insight into the individual glycemic variability (GV) of a person with diabetes. The more detailed insight provides actionable information for the individual diabetes management [16]. As a result, the more stable GV can lead to a decrease in oxidative stress, inflammation, and associated complications such as macrovascular damages [17]. In addition, the use of CGM during and after physical activity can prevent hypoglycemic episodes, or more precise decisions on insulin adjustments or additional glucose uptake if needed can be made [18]. With the aid of visualization of short-term improvements in glycemic control, an increase in motivation to maintain the increased exercise behavior can be achieved. Motivation and a sense of self-efficacy in particular are the most important key factors in order to maintain lifestyle changes in the long-term [12].

Long-term effects can already be observed. In one study, using CGM systems for three days per month for 12 weeks resulted in increased physical activity and an additional 1% reduction in HbA1c in people with previously poor glycemic control [19]. Another study demonstrated similar effects of CGM use such as increased physical activity and a 1.2% reduction in HbA1c [20].

### 2.1. Increase in TIR for People with T2D during Training through the Use of CGM 

CGM can lead to improvements in time spent in the euglycemic range which is also known as TIR [15,18]. It can be seen as an advantage of CGM that during the training not only single measured values can be monitored. Trend arrows can be used to interpret and predict the further course of the glucose levels [21,22]. The study of Beck et al. also could show improvements in HbA1c due to the use of CGM in comparison to usual care in insulin-dependent people with T2D. The mean improvements in HbA1c levels in the treatment group were 0.8% versus 0.5% in the control group at the study end. Even HbA1c improvements of 0.3% were seen without medication adjustments [15]. The benefits of CGM to predict the following glycemic courses and also taking into account the influences of sports lead to a recommendation of CGM use, especially in physically active people with diabetes.

### 2.2. Optimized Therapy and Documentation through the Use of CGM

A study showed that CGM data in conjunction with previously used insulin adjustment algorithms can lead to increased improvements in glycemic control [15]. Through the interpretation of trend arrows of CGM proactive actions in connection to physical activity can be decided. This can be, for example, additional glucose uptake before a hypoglycemic episode appears due to the expanded physical activity [18]. The alerts of CGM can be set accordingly to prevent adverse events. Various CGM systems which can send an alarm are available in the market [18].

Courses and trends visualized with a CGM can give a prospective view of the interstitial glucose courses and the velocity of the courses. This can be used to predict the possible further course. Additional influences such as sports or acute illness can be taken into account [18,22]. The optimized documentation through CGM data can give a comprehensive view of the ambulatory glucose profile of the individual using CGM. These data can be given out in visualized overviews (e.g., weekly summaries). The generated data and also visualization allows us to optimize and finetune the individual’s diabetes management. Physicians together with people with diabetes can base their therapy decisions on the generated data pool and the associated experiences [22].

It can be recommended for physically active people with diabetes to take trend arrows into account for therapy adjustments, especially for short-term adjustments due to physical activity. Possible adverse events can be prevented and the joint evaluation of the visualized glucose courses together with the physician makes it possible to evaluate the effects of the training.

### 2.3. Relevance of CGM for Prevention of Hypoglycemia

Fear of hypoglycemia is one of the strongest barriers that prevent people with T1D from incorporating more physical activity into their daily lives [13,14,18].

Hypoglycemia may occur during physical activity and in the first hours afterwards due to a mismatch between glucose production and utilization [23]. Cumulatively, insulin injection, the type, intensity, and duration of exercise, and the time interval between activity and the last food intake influence glucose homeostasis [24,25]. In metabolically healthy people, at the beginning of aerobic, light to moderate physical activity, there is an increase in glucagon levels and a simultaneous decrease in insulin release. In contrast, during more intense exercise sessions, that are transitional between aerobic and anaerobic levels, insulin release initially decreases and rapidly increases again during the early recovery phase. This increase occurs to balance counter-regulatory hormones and metabolites [25,26]. These fluctuations during intense exercise sessions can lead to high GV and hypoglycemia in people with diabetes. Even mild to moderate exercises can increase insulin sensitivity for the following 11 to 16 h and, in combination with metabolic counter-regulatory mechanisms, lead to late-onset or nocturnal hypoglycemia. To avoid or shorten the duration of hypoglycemic episodes during and within the 2 h period after physical activity, it is recommended that people with T1D complete strength training sessions before aerobic endurance training sessions [27]. A study showed that completing the weight training session before endurance training, as opposed to the opposite order, resulted in fewer drops in glucose levels during exercise, fewer hypoglycemic episodes, and less frequent need for carbohydrate supplementation. However, if hypoglycemia occurred within 12 h after exercise, its duration was shortened, and its severity was reduced [27]. The order of training sessions is also thought to influence the occurrence of nocturnal hypoglycemia [27]. A dependence of hypoglycemia on the intensity of the weight training sessions (from light to moderate) could not be observed. In the study to which this observation refers, no hypoglycemia occurred at all in the recovery phase after weight training [28]. Incorporating short sprint sessions of 10 s before or after workouts can also prevent hypoglycemia immediately after exercise [29]. Sprints after high-intensity training can’t be recommended as the blood glucose levels tend to be more stable during the first hour after training [30]. In order to quickly detect the glycemic changes that occur during phy- sical activity and its recovery phase, individual blood glucose checks would have to be performed very frequently. These can be replaced by CGM so that changes during the sports session can be observed in real time [26].

Thus, the use of CGM systems by people with diabetes can help to detect glycemic fluctuations more quickly during the exercise session, as well as prevent the risk of hypoglycemia and treat hypoglycemic episodes immediately [26,31]. The use of CGM and its progress curves offers the possibility to avoid hypoglycemia or to react to it at an early stage, especially for people with T1D who are regularly physically active [32]. Based on the measurements and the alarm limits set, well-founded decisions can be made on glucose uptake for hypoglycemia prevention or treatment [32]. These alarm limits have to be orientate the expected delay between the measured interstitial glucose and the blood glucose level [18].

It can be recommended that the order of the training should be: at the beginning of the training session, weight training followed by endurance training. This order can prevent hypoglycemic events and nocturnal hypoglycemia in the first hours after training sessions. CGM utility makes it possible to assess the risk of their occurrence and/or makes quick interventions as additional glucose uptake possible. Alarm limits should be set and checked regularly.

## 3. Improved Compliance and Motivation for Physical Activity after CGM Use 

**Figure 1:**
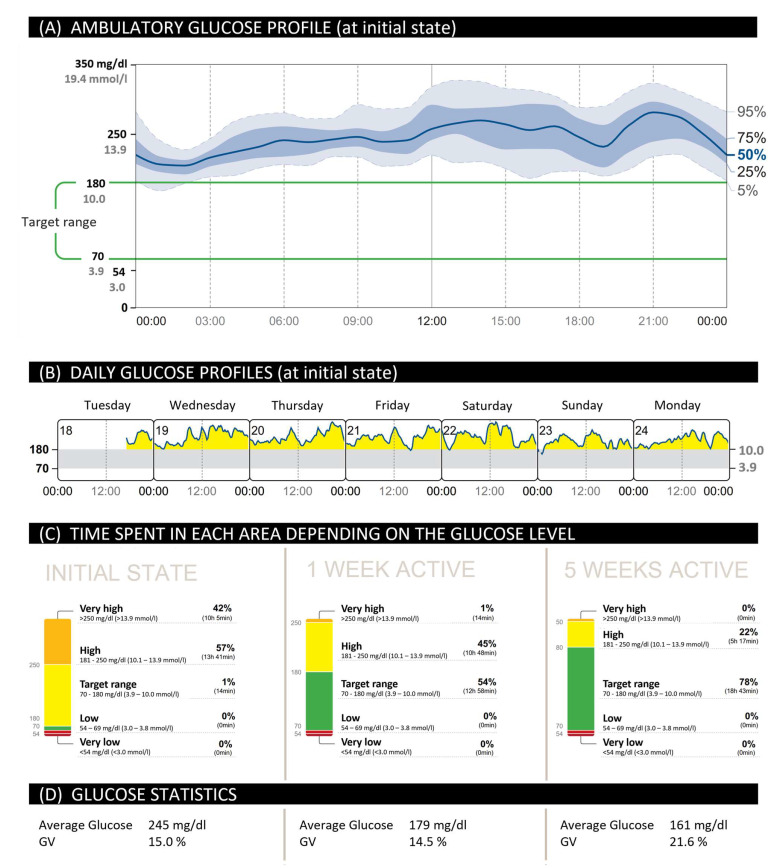
CGM data report of a 54-year-old German man with type 2 diabetes (T2D)—a real-world case study from February 2020 (adapted to Brinkmann et al. [33]). Ambulatory glucose profile (**A**) and daily glucose profiles (**B**) before regular physical activity. Data on time in range (TIR) (**C**) and glucose statistics (**D**) are indicated for three different time points—at the patient’s initial state (left), as well as after one (middle) and five weeks of physical activity (right). GV: glycemic variability.

In the following real-life case study from February 2020, one can appreciate the improvement of the glucose levels of a person with T2D after the use of a CGM system (Figure 1) [33]. A 54-year-old man with a BMI of 32 kg/m^2^ was diagnosed with T2D in 2004. Before the implementation of the CGM system, his last HbA1c level was measured at 8.2%. He was treated with oral anti-diabetica and had no comorbidities. 

The CGM data assessment covered a 7-day period, which was shorter than the re- commended two weeks. Nonetheless, at 92%, data quality was very good. 

The CGM data were analyzed in form of an ambulatory glucose profile (AGP). AGP enables the visualization of glucose values from the reporting period, with the median and other percentiles displayed as if they related to a single day. Thus, the AGP of the patient can easily display the hypoglycemic risk and GV. In this case, the GV between midnight and 12 pm was low but increased in the afternoon and evening hours (Figure 1A). 

Additionally, the CGM system provides an overview of the patient’s glucose levels on a daily basis (Figure 1B). The everyday profile relates to the period from 12 a.m. to 12 a.m., with the date indicated in the upper left-hand corner of each daily profile. The graph showed the patient’s glucose levels were predominantly above the target range.

The quantification of the data is summarized in panels C and D of Figure 1. At the initial state and before regular physical activity, the patient’s glucose levels were above the target range 99% of the time. This percentage is considered very high. Moreover, the average glucose (245 mg/dL resp. 13.6 mmol/L) was considerably increased. No low glucose events were observed with 0% of the time spent below the target range. Therefore, hypoglycemia prevention was not prioritized. 

Based on these data, the patient’s diabetes team recommended lowering his elevated glucose levels through regular exercise. Due to his obesity, movements that are easy on the joints and CV system, such as slow cycling or walking, were recommended. His diabetes team supported him by motivating him with calls once a week. His results after one and five weeks of physical activity, respectively, are also provided (Figure 1, middle and right section of panels C and D). With regular cycling, work in the garden, or strolls for at least an hour a day, the time within the target range was increased from 1% to 78%. Consequently, the sharp glucose rises, as well as the time above range, the number of hyperglycemias, the average glucose value, and the Glucose Management Indicators were reduced. The patient was excited about the results and was keeping his glucose levels under better control in the future. This particularly motivated him to continue integrating physical activity into his daily routine.

## 4. Importance of Exercise in Everyday Life in Prediabetes and Diabetes

Physical inactivity and a sedentary lifestyle can influence the course of various di-seases such as diabetes negatively [34]. A meta-analysis showed that the relative risk for total mortality can increase by a factor of 1.22, for CV mortality by a factor of 1.15, and for the occurrence of T2D by a factor of 1.91 due to a predominantly sedentary lifestyle [35]. In contrast, physical activity has a positive pleiotropic effect. With regard to diabetes and frequently associated metabolic syndrome, the positive effects of physical activity can be substantiated with the highest level of evidence, class Ia [14]. Even small changes in everyday physical activity, such as taking a short break every hour while moving, can have a positive impact on body weight. Thus, up to 132 additional kilocalories can be burned during an 8 h workday [36]. In a study, walking for 20 min at a self-selected pace after dinner was shown to be effective in lowering glycemic impact in older people with T2D compared with walking before dinner or no physical activity [37]. However, the positive training effects are lost within a short time when regular activity is paused or stopped, even if no changes in cardio-respiratory fitness, body weight, and body fat percentage are yet noticeable. After ten days of no exercise, plasma glucose and insulin resistance (IR) return to the level of the untrained state [38].

In the following, we will discuss in more detail the different, provable positive influences of physical activity on glycemic control, GV, postprandial glucose progression, nighttime glucose progression after exercise, the different effects depending on the time of day of the exercise session, and the reduction in IR.

### 4.1. Influence on Glycemic Control

Regular physical activity leads to an improvement in glycemic control. A total of 150 min of physical activity per week recommended in the American Diabetes Association (ADA) guidelines can reduce HbA1c in the long term [39,40,41]. Studies showed that weight training can reduce the HbA1c by about 0.6% [40]. Increasing the duration of exercise above the recommended 150 min per week results in an even greater reduction in HbA1c of 0.89% [40]. The combination of weight and endurance training leads to a greater effect than weight training alone. A meta-analysis showed that on average endurance training reduced HbA1c by 0.73%, weight training by 0.57%, and the combination of both by 0.51% over the defined period [40].

Improving HbA1c can reduce the risk of comorbidities [42]. For example, lowering HbA1c results in a 14% reduction in the risk of myocardial infarction. The risk of diabetes-associated death can also be reduced by 21% by lowering HbA1c [10]. The risk of microvascular complications can be reduced by 37% with a 1% HbA1c reduction [10]. Overall, good glycemic control with HbA1c levels < 7% can reduce the risk of long-term complications by up to 76% [10,43].

To increase glycemic control and to decrease the risk for diabetes-related complications of an individual with diabetes, the authors recommend a combined training of weight and endurance sessions for 150 min during the week.

### 4.2. Influence on Glycemic Variability

One study was able to show that high-intensity physical activity can positively influence GV in people with T2D. However, this did not reduce the mean CGM glucose level [44]. Furthermore, this study showed that fluctuations in glucose levels may be more harmful than a constant high and stable mean glucose level [44]. The fluctuations may have effects on endothelial function as well as oxidative stress, which promote CV complications in the diabetes [17]. Thus, a reduction in GV through physical activity may be of higher importance than a reduction in mean glucose level [44].

Therefore, the courses and glycemic fluctuations in relation to physical activity should be considered and evaluated in defined periods, e.g., daily or weekly summaries of the glucose courses.

### 4.3. Influence on Postprandial Glucose Levels

Persistent postprandial hyperglycemia is the main cause of oxidative stress and HbA1c elevations in people with T1D [45] and in those with T2D [46]. In a study, CGM-measured postprandial glucose courses were compared with respect to the influence of physical activity before, immediately after, or 30 min after breakfast on postprandial glucose courses. Low- to moderate-intensity physical activity immediately after breakfast was shown to decrease glucose uptake and GV. Even standing for 30 min immediately after a meal had a slightly positive effect. In contrast to that, physical activity before or 30 min after breakfast showed no positive effect on GV. Often it is not possible to do physical activity directly after meal intake. In such situations, at least an attempt should be made to change to a standing position [47].

In line with the previous study, an additional study on the effects of a twenty-minute training of moderate intensity after dinner showed that the 2 h postprandial glucose spike, the mean glucose level, and the peak glucose level can be reduced in people with T2D. The occurrence of nocturnal hypoglycemia was not increased after the training [48].

According to the positive effects and the not appearing negative consequences of post-meal and post-dinner training, a short training session after each meal can be recommended. If training is not possible directly after meal uptake, at least getting in a standing position can be recommended.

### 4.4. Night Course of Glucose Level after Exercise

A study showed that moderate-to-vigorous intensity physical activity in the late afternoon or evening for 30 min can increase the risk for the appearance of nocturnal hypoglycemia by up to 43% or 31% in people with T1D [49]. Nocturnal hypoglycemia after exercise results from the muscle glycogen stores being replenished during the recovery phase. In the period between midnight and 4:00 a.m., there is an increased hypoglycemia risk for people with T1D after endurance training sessions [50,51]. Adolescents with T1D in particular must compensate for this with increased glucose intake to remain normoglycemic [27]. Using CGM enables us to analyze the direct correlation between physical activity and followed hypoglycemia in people with T1D [49]. It can help to prevent hypoglycemic episodes or make quick therapy adjustments as additional carbohydrate uptake [52].

To avoid nocturnal hypoglycemia due to physical activity, it can be recommended to consider additional glucose uptake according to the recommendations of the ADA (see Table 1). Additionally, the alarm limits of the CGM during the night after a training session should be narrowly set.

### 4.5. Different Effects Depending on the Time of Day When Training Was Executed

Exercise sessions, depending on their type and duration, can induce hypo- or hyperglycemia in the early morning before taking breakfast, under intensive insulin therapy with a basal insulin. This may be attributed to the low injected basal insulin concentration in the early morning during this therapy. Physical activity performed after breakfast may react together with the first injection of basal insulin in the morning before breakfast. Du-ring the exercise session after a meal, glucose from the blood is used and consumed as an energy source, so that postprandial hyperglycemia can be avoided [53]. However, it is also possible that hypoglycemia occurs due to inhibited release of glucose by hepatic glycogenolysis [53,54].

Insulin adjustments in case of physical activity in the morning should be considered according to the ADA recommendations to avoid hypoglycemic episodes (see Table 2).

### 4.6. Reduction of IR

It is well known that T2D is predominantly associated with IR. IR is understood as the presence of hyperinsulinemia coupled with impaired glucose tolerance. The presence of IR can be used as a negative biomarker for T2D, which represents poor metabolic constitution [12,55,56,57]. The pancreas can still have a compensatory effect in the early stages of IR. It runs this by increasing insulin secretion into the bloodstream to overcome the defects in peripheral insulin action. The β-cells hypertrophy in response. In the fasting state, basal compensation is thereby sufficient and glucose levels remain within the normal range. After food intake, when glucose is rapidly absorbed from the intestine, a relative insulin deficiency occurs due to insufficient compensation [58].

Obesity can be seen as one of the main causes of IR [58]. The combination of a diet high in fat and carbohydrates, and low physical activity in conjunction with a genetic predisposition can result in IR and thus in T2D [58].

Body cells’ IR is characterized by decreased insulin-stimulated glucose uptake in skeletal muscle cells and adipose tissue [55,59]. The intensity and frequency of physical activity result in different increases in the amount and rate of glucose uptake in skeletal muscle [55,59]. Both endurance and weight training can increase glucose transport into cells [55]. Furthermore, increased physical fitness is associated with a high level of insulin sensitivity and thus increased insulin action [55,58]. Skeletal muscle contraction can increase energy expenditure by 8 to 10 times resting energy expenditure [54]. Reduced IR may also increase the overall life expectancy [60]. The lowering of IR and the resulting improved insulin sensitivity of muscle cells need not be accompanied by body weight loss. However, studies have shown that there was no weight loss despite improvement in insulin sensitivity [61,62,63].

For assessment of the efficiency of the diabetes intervention tool physical activity/sports, not only the actual weight loss but also the IR should be seen as a parameter. The possible decrease in the IR through regular physical activity has the potential to increase life expectancy. Due to higher insulin sensitivity, the number of insulin injections can be adjusted.

### 4.7. Long-Term Effect of Exercise on Glycemic Control

A study on people with T2D on the effects of interval walking training vs. continuous walking training in an observation phase of two weeks showed significant positive effects on the fasting glucose in the intervention group. Mean glucose levels did not change. Regular walking training resulted also in significant reductions in the CGM measured mean and maximum glucose levels and the individual’s time spent in hyperglycemia. Independent of changes in the individual’s body composition and physical fitness, performed interval walking training led to an increase in glycemic control and GV and showed additional beneficial positive effects on the total cholesterol and triacylglycerol [64]. Due to these positive long effects, interval training can be recommended.

### 4.8. Effects of Extreme Conditions as Low Temperature and High Altitude on CGM Measured Physical Activity

Physiological physical activity at high altitude leads to an increased glucose and insulin level in metabolically healthy people [65]. After acclimatization, the glucose and insulin levels adapted to levels which can be seen at physical activity at sea level [66]. People with diabetes can acclimate to high altitudes but have defective counter-regulatory mechanisms against the increase in glucose. Thus, it is difficult to predict the actual insulin requirement and glycemic control during high-altitude physical activity [65,67].

Elevated counter-regulatory hormones against the occurring hypoxia at high altitude can influence glycemic control, especially if people with diabetes get acute mountain sickness (AMS) [67]. Physical activity at high altitudes can increase the glucose level and thus the required amount of insulin. This can occur despite the increased energy expenditure [65,68]. Due to these difficulties for stable glycemic control, glucose levels should be monitored frequently. Since the accuracy of capillary blood glucose monitoring can be lower in an environment of high altitude and cold temperature [67], CGM systems can be re-commended during physical activity in this environment [65,67].

In a study on the reliable measuring of CGM and glycemic control during physical activity at low temperature and high altitude, no performance problems were reported and no cases of significant glycemic decompensation were seen. The study examined people with T1D with CGM and/or insulin pump systems during hiking at 5000 altitude meters. As all of the participants reported symptoms of AMS (e.g., headaches, nausea, dizziness), it was assumed that moderate hyperglycemic episodes were in conjunction with AMS and not to the inaccurate measuring of CGM or the insulin delivery of the insulin pumps [69]. AMS also promotes raising the activity of the sympathetic nervous system and cortisol levels. High cortisol levels led to low insulin sensitivity and can evoke hyperglycemia [67]. The symptoms of AMS can make it difficult to realize hypoglycemia [68]. There is no difference in frequency of AMS between people with T1D and metabolically healthy people [65,68].

Accurate CGM systems which are not highly affected by extreme conditions such as low temperature and high altitude can be recommended to address the higher risks for glycemic fluctuations and GV in this environment.

### 4.9. Exercise in Everyday Life for Prediabetes

Prediabetes is defined as subclinical impairment of fasting plasma glucose levels, impaired glucose tolerance/insulin resistance, or both [70]. Prediabetes is considered as a risk factor for developing T2D. A distinction is made between three prediabetic phenotypes. Phenotype 1 is characterized by impaired glucose tolerance (IGT) without impairment of fasting plasma glucose (FPG). The increase in peripheral insulin resistance in skeletal muscle can only be detected by an oral glucose tolerance test (OGTT). Phenotype 2 is impaired fasting glucose (IFG) with impairment of FPG. This phenotype reflects hepatic insulin resistance and is more easily diagnosed from a fasting blood sample. Phenotype 3 is a combination of IGT and IFG [71].

According to the ADA, prediabetes is present at an FPG level of 5.6–6.9 mmol/L, and/or a 2 h OGGT at an OGTT level of 7.8–11.0 mmol/L and is considered as diagnosed at an HbA1c in the range of 5.7–6.4% (38–46 mmol/mol). In contrast, the World Health Organization (WHO) specifies an FPG of 6.1–6.9 mmol/L and a 2 h OGGT of 7.8–11.0 mmol/L, or a combination of both, as the diagnostic value. There is no evaluation of the HbA1c [70,71].

Lifestyle interventions such as dietary adjustments and exercise are considered first-line therapy for prediabetes [71]. Lifestyle interventions such as physical activity can delay the progression from prediabetes to T2D. However, the positive effects of lifestyle interventions are particularly pronounced in older people. Furthermore, it was shown that lifestyle interventions have a greater effect than drug therapy in the metformin [72]. Another study showed that the combination of dietary change and physical activity was able to reduce the incidence rate of T2D within 6 years in people with prediabetes to 46% compared to the control group without intervention with 67.7%. In the group that used only the physical activity intervention, the incidence was reduced to 41.1% [73]. In the long term, if prediabetes is present, the goal is to obtain permanent weight loss through lifestyle interventions and thus prevent the onset of T2D [71].

## 5. International Recommendations on Physical Activity of People with Diabetes in Interaction with CGM

In a systematic review from 2020 on the recommendations for exercise in subjects with T2D that was valid at that time, fifteen different recommendations were examined for consistency. Ten out of fifteen of the reviewed recommendations recommended aerobic endurance training at least three days per week. Eight out of Fifteen advised strength training. Combined training was recommended in three of the fifteen recommendations and supervised training was advised in two of the fifteen recommendations. The recommendations differ greatly with regard to the aspects studied [74].

In the following sections, a brief overview of the recommendations especially focused on T2D of the ADA and the American College of Sports Medicine (ACSM), national health care guidelines and practice recommendations of the Deutsche Diabetes Gesellschaft (DDG) will be given. Furthermore, the question of an optimal number of steps per day for a long-term healthy lifestyle will be discussed.

### 5.1. ADA and ACSM Recommendations

#### 5.1.1. Generally Applicable for T1D and T2D

The ADA recommends increasing daily activity in general, but especially for people with T2D. Long periods in a sedentary position should be alternated with periods of physical activity every 30 min. Prolonged periods of low activity lead to poor glycemic control, increase further metabolic risks and are associated with increased mortality rates [21]. The recommendations of the ACSM and the ADA apply in addition to the recommendation to expand structured physical and incidental activity [21,75]. In principle, for asymptomatic people with diabetes, no medical examination is needed before starting low or moderate intensity physical activity, if it does not exceed the requirements of brisk walking or daily living [21]. For most adults with diabetes, at least 150 min of moderate to vigorous intensity physical activity is recommended weekly. This active time should be performed over at least three days a week. There should be no more than 2 consecutive days without activity. Shorter sessions of at least 75 min per week of higher intensity or interval training may be sufficient for younger and more physically fit individuals [21].

Exceptions for certain people with diabetes and peripheral neuropathy and without acute ulceration, moderate strength training sessions are recommended by both the ADA and the ACSM [75].

With 2–3 training sessions per week on non-consecutive days, strength training exercises should be integrated [75]. Adults with diabetes should do resistance exercises 2–3 times a week on non-consecutive days. For the elderly, flexibility and balance exercises are recommended 2–3 times a week. For example, yoga and tai chi can be used to improve one’s flexibility, muscle strength, and balance depending on individual preferences. Balance exercises have the additional effect of preventing falls of elderly people [21,75]. However, this does not result in any positive effects on insulin action, glucose control, or body composition [75]. People with prediabetes or diabetes are encouraged to increase their daily incidental exercise. To gain more health benefits from exercise programs, participation in supervised exercises is recommended over unsupervised programs. For people with T2D, caution should be exercised if blood glucose levels are above 300 mg/dL (16.7 mmol/L) when exercising. To be able to exercise, they should feel well and hydrated [75].

#### 5.1.2. Type 2 Diabetes

For people with T2D, daily physical activity or, in the case of breaks between exercise sessions, a maximum break of 2 days, is recommended to enhance insulin action. Adults should use endurance and strength training if possible. Structured lifestyle interventions are also recommended. These include at least 150 min of physical activity per week and dietary changes that should lead to a weight loss of 5–7%. In addition to endurance training, people with T2D should engage in moderate to vigorous strength training at least 2–3 days per week. These interventions can prevent or delay the onset of T2D in people with increased risk for prediabetes [21,75].

#### 5.1.3. Recommendations to Prevent Adverse Events due to Physical Activity of People with Diabetes

To avoid hypoglycemia during physical activity, adjustments in carbohydrate intake and insulin administration should be made. It is also recommended to include short sprints and to perform weight training before endurance training. The duration of a training session should also be kept in mind. To reduce the risk of nocturnal hypoglycemia after physical activity, the basal insulin dose can be lowered or the intake of small snacks before bedtime and/or CGM can be completed. A targeted skip of insulin before an exercise session can be a trigger for hyperglycemia. Hyperglycemia as another adverse event due to prior exercise is more common in people with T1D. This can be prevented by adjustments in the amount of insulin or a less intense cool-down phase. In the presence of hyperglycemia and elevated blood ketones exercise is not recommended [21]. Medications other than insulin may also increase the risk of exercise-induced hypoglycemia. The doses of these medications may need to be adjusted as well as the insulin [21].

For people with diabetes who are untrained in routinely exercising, it can be recommended to start their training sessions with higher glucose levels than the trained, to avoid hypoglycemia [76].

Elderly people with diabetes or people with autonomic neuropathies, CV complications or lung diseases should avoid exercising outdoors on very hot and/or humid days to prevent heat-induced diseases. Exercise training should be completed at a pace that is not too fast in order to minimize the risk of injury, e.g., by falling [21].

### 5.2. German National Health Care Guidelines and Practice Recommendations of the DDG

In its practice recommendation “Diabetes, Sport and Exercise”, the DDG refers to the recommendations of the ADA. Even light forms of exercise “(...) according to the motto ‘running without puffing’ (...)” are considered positive for people with diabetes. Furthermore, for the treatment of obesity, it is recommended to increase the exercise time to 200 to 300 min per week in order to achieve an energy deficit of 500 to 750 kilocalories per day for weight reduction [54]. Due to individual differences in blood glucose levels after physical activity, the DDG recommends increased and regular blood glucose measurements during the activity and up to 12 h after it, if sporting activities have been newly integrated into everyday life. The use of a sports diary is also recommended, in which the glucose courses, insulin administration, the injection–exercise time interval, the ingested additional carbohydrates, and the characterization/type, intensity, and duration of physical activity are documented. This protocol can be included in therapy optimization [54].

CGM systems can be helpful to assess metabolic responses during and after exercise. In the case of physical activity, it should be noted that the CGM measuring systems must be covered so that it is waterproof. An adjustment of the alarm limits should be made before the start of physical activity. Adjustments to automatic insulin dosing (AID) systems may also be necessary [54].

Even prolonged and intense physical activity, such as half marathons, can be safely completed by people with T1D using closed-loop systems and a Do-it-Yourself Artificial Pancreas System. In this study, it has been demonstrated that avoiding hypoglycemia as well as hyperglycemia was possible by setting temporary closed-loop targets [77]. 

Ergometry, including lactate testing or spiroergometry under different training intensities, can be used to determine individual metabolic limits and pulse rate. The meta-bolism in the aerobic and anaerobic range can be determined and the resulting outcomes can be included in the training recommendations [54]. To prevent and treat quickly hypoglycemia, the DDG recommends carrying an “SOS sports kit” with foods that act quickly on the blood glucose, such as glucose, during physical activity. Sports partners and other known people present during training should be informed about the risk of hypoglycemia and possible countermeasures [54]. For hypo- and hyperglycemia prevention during and after physical activity, the DDG gives strategies for adjusting the amount of insulin or the intake of additional carbohydrates. The factors listed in Table 1 and Table 2 play a role in the insulin adjustments and the additional intake of carbohydrates that may be necessary.

### 5.3. How Many Steps Can Be Recommended?

The step index for a measured number of steps per day according to Tudor-Locke and Bassett provides for the following gradations in the classification of the intensity of step units. A step count of fewer than 5000 steps per day is described as “mainly sedentary”. A daily step count between 5000 and 7499 steps is described as “low activity”. A total of 7500 to 9999 steps per day are considered “somewhat active”, 10,000 to 12,499 steps per day are considered “active” and over 12,500 steps per day are considered “very active” [78].

Activity trackers and accelerometers provide the opportunity to accurately monitor and document an individual’s physical activity. This allows medical professionals and other healthcare professionals, to share the data generated, as an aid to medical decision-making [28]. In addition, activity tracking can be incorporated into decision-making when calculating daily insulin requirements [11]. Various suggestions exist for recommendation on the daily physical activity requirements. A study review from 2011 summarized the recommendations for the number of steps for moderate to vigorous physical activity as follows: 8000 to 11,000 steps per day were recommended in the studies reviewed. It should be noted that other activities, e.g., of the upper body, can not be recorded by pedometers in some cases. The recommendation also takes into account that there is not a constant activity level every day. It follows that the average daily step count over a week should be between 7100 and 9000 steps. At least a proportion of these steps (about 3000 daily for a total weekly step count of 15,000) should be taken at a minimum intensity of at least 100 steps per minute per exercise session of at least 10 min. The various recommendations range from 4000 to 18,000 steps per day.

### 5.4. Risk for Complications Depending on the Number of Steps per Day

It was shown that a step count below 5000 steps per day increases the prevalence of CV risks. Another study showed that the general health risks such as the development of metabolic syndrome can be positively influenced overall by the daily step count [79]. Particularly in women, the positive side effect of a 50% reduction in the pre-valence of depression was also shown with the daily moderate exercise of over 7500 steps per day. In men, this effect occurred with a daily step count of over 12,500 compared to a step count of under 5000 daily steps [80]. An appropriate number of steps in the range of the recommendation can contribute in particular, but not exclusively, to long-term health maintenance and sufficient daily physical activity [78].

## 6. Exercise on Prescription

Physical activity as an intervention tool in diabetes management can be used both: preventively and therapeutically [14]. Light to moderate forms of exercise such as stair steps, doing the daily shopping on foot, fast walking or Nordic walking have positive effects on the entire body. Exercise as a causal therapy concept can be individually adapted and prescribed according to the personal preferences and life circumstances of the person with diabetes [81]. The prescription can be remunerated in Germany according to the German Prevention Act, but so far there is no figure according to the Uniform Assessment Scale (“Einheitlicher Bewertungsmaßstab”). Individual counseling should precede the prescription of personalized physical activity therapy treatment. The counseling on physical activity should take place in connection with dietary adjustment counseling and can thus lead to a 0.58% lower HbA1c [40]. Counseling sessions can take place and be billed, for example, within the framework of sports medical care or a disease management program. They can be directly oriented towards the exercise recommendations of the individual professional associations [81].

Studies have shown that collaborative goal setting between people with diabetes and their caregivers led to improved self-management skills and a strengthened relationship with the doctor. This in turn led to better glycemic control [12]. The jointly selected exercise programs should be individually adapted to the person with diabetes. Physical and psychological overload resulting from the exercise program may inevitably lead to program discontinuation. Especially within the first six weeks after the start of new exercise programs, this is of high importance [54]. A dose–response relationship with physical activity is present, but not linear [14]. Increasing the number of exercise sessions does not necessarily increase the positive effects to the same extent. The effect of defined activity units also has different effects on the individual person with diabetes [14].

In general, exercise should be started at a low intensity and gradually increased. If necessary, a check-up by a sports physician can be carried out to monitor the progress. Physical activity interventions can be adapted for the elderly, which should be supplemented by regular everyday activities such as gardening [54,81]. Training sessions should be completed at a frequency of three to four times a week with a duration of 30 to 50 min per session. Suitable types of training include swimming, dancing, or cycling. In addition, strength training should be integrated two times a week. [14]. Through the exercise on the prescription concept, people with diabetes can also be referred directly to appropriate, quality-assessed, or certified sports groups and clubs. In addition to prescribing exercise on prescription, sports physicians have the option of prescribing physical activity via an Exercise Prescription for Health (EPH) prescription sheet, approved by the European Fe-deration of Sports Medicine Associations (EFSMA) [14].

The subsequent evaluation meetings can take place regularly at intervals of three to four months. Here, any necessary training adjustments and therapy adherence should be checked [12,14]. Guided training, e.g., in sports groups, can lead to stronger positive effects of HbA1c, BMI, and waist circumference [82]. Additionally, guided training by a fitness expert or physical therapist can increase effectiveness and safety from injuries [21]. To reduce the risk of injury, the ACSM recommends incorporating warm-up and cool-down phases [83].

## 7. Different Moderate Forms and Types of Physical Activity Which Can Be Easily Integrated into Everyday Life and Strategies for Better Integration

Physical activity can be classified as aerobic, mixed aerobic/anaerobic, and anaerobic in terms of its activity form [54].

### 7.1. Anaerobic/Untrained und Aerobic Sports

Sports can lead to a one hundred times higher oxygen consumption. The free radicals which occur through the increased oxygen demand can have negative outcomes on di-seases. Untrained or anaerobic sports can have higher negative effects on oxidative stress driven by the activity than aerobic sports. Aerobic training tends to have a longer duration with lower intensity than anaerobic training. The effects on the occurrence of oxidative stress are influenced by the intensity and duration of the exercise. A study showed that the duration at which oxidative stress increases can vary between 90 and 120 min [84]. It can be concluded that the intensity of an exercise as a co-factor for oxidative stress should be closely looked at [84].

To make an easy distinction if an individual’s training is aerobic or anaerobic during the physical activity, the following advice can be given. Being able to talk or sing during the training shows that there is an adequate supply of oxygen, and the activity is on an aerobic level.

### 7.2. Integration of the BORG and OMNI Scale for Intensity Determination

As physical activities even with low or moderate intensity show positive effects on glycemic control, scales for subjective intensity determination should be integrated in the training routine. Both scales, the BORG (6–20) [85] and the OMNI scale (0–10) [86], rate the level of exertion and are indirect markers of the blood lactate response and stress to the carried out exercise [87]. Through BORG the level of exertion can be described from 6 as “no exertion” to 20 as “maximal exertion” and through OMNI the difficulty level can be described from 0 as “extremely easy” to 10 as “extremely hard”. Supportive visualization aids for rating are available [87].

In the following, reference is made to some forms of exercise of light to moderate intensity that can be easily integrated into everyday life, as well as to strategies for integrating them more easily into everyday life.

- Stroll/Walking

Walking is defined as aerobic physical activity. The heart rate is around 100 heartbeats per minute [53]. Studies showed that walking after food intake in the morning has a significant lowering effect on blood glucose levels [53]. In particular, for people with well-controlled T1D and administered basal bolus insulin, it is recommended that the exercise session of walking be performed after the meal. This can significantly reduce postprandial glucose trajectories and lead to an improvement in glycemic control [53].

- E-Bike

In recent years, the e-bike has become a very popular means of transportation and sports equipment. In 2012, 31 million e-bikes were sold worldwide [88]. Due to the higher intensity and therefore higher heart rate that can be achieved when cycling compared to running, this can result in better fitness [89]. Cycling in itself has been shown in studies to have positive effects on the risk of CV disease, CV fitness, and overall morbidity and mortality [90,91]. A study showed that cycling for 3.5 h per week resulted in a 23% reduction in the risk of CV death. Cycling more than 3.5 h per week resulted in a reduction of 34% [92]. Another study showed the positive influences on body weight in pre-menopausal women. Already 5 min a day had positive effects. The duration of cycling had a direct influence on the amount of weight loss [93]. Additionally, middle-aged people who engage in daily physical activity by bicycle perceive the positive effects themselves [94]. E-bikes have an additional assistance function, which also enables older and less fit people to be assisted in overcoming obstacles that are perceived as too strong, so that they can also pursue physical activities by bicycle. E-bikes can be integrated very well into everyday life and can also be used for longer distances, e.g., for shopping. [89].

Other physical activities that can be easily integrated into everyday life are changes in daily habits such as: taking stairs steps instead of elevators or escalators, walking the last subway stop before the destination, running small errands on foot or by bike, or iro-ning in a standing position.

## 8. The Importance of Physical Activity for People with Pre-Existing Conditions—What Should Be Considered in the Case of Pre-Existing Conditions and Which Preventive Examinations Are Necessary? When Should a Sports Physician Be Consulted?

Physical activity poses several health risks for people with diabetes. These include acute complications such as cardiac events, hypoglycemia, and hyperglycemia [21]. Low- and moderate-intensity physical activities have only a low risk for people with T2D, in contrast to people with T1D. For people with T2D, hypoglycemia is the only more common adverse event [21].

Before taking up physical activity, limiting or contraindicating secondary and pre-vious illnesses should be clarified. This often requires preliminary examinations by a sports physician [14]. According to the ADA and the ACSM, pre-existing CV diseases are not to be seen as fundamental contraindications for physical activity [75]. Before taking up regular physical activity, people with medical abnormalities and people aged 35 and older should always have exercise echocardiography (ECG) to give an indication of pre-existing CV conditions [14].

The ACSM has published a model for medical screening before starting regular phy-sical activity. This is based on the level of physical activity prior to starting the new exercise, the intensity level, the pre-existence of CV, metabolic, and renal symptoms, and signs of disease [83]. The ACSM recommends that anyone with diabetes who would like to incorporate new physical activities of any intensity into their daily life should have a medical screening before. However, the screening does not need to include a risk assessment [83]. For people with diabetes who do not show symptoms of pre-existing conditions, no benefit of risk reduction of adverse cardiac events due to additional medical screening has been shown so far [95,96]. Furthermore, the ACSM recommends that people with diabetes are adequately trained to recognize warning signs and symptoms such as chest pain and palpation [83].

People with cardiac autonomic neuropathy and diabetes should be pre-screened and receive physician approval and possibly an exercise test before starting any new physical exercises. These exercises should be prescribed with heart rate framework prescriptions if possible [75]. When engaging in physical activity, especially in the presence of peripheral neuropathy, attention should be paid to comprehensive foot care, including daily inspection of the feet, and the use of appropriate footwear is recommended to prevent and detect sores or ulcers early. Walking at a moderate pace does not increase the risk of ulceration or re-ulceration in peripheral neuropathy [75].

Physical activities that greatly increase intraocular pressure and the risk of hemorrhage should be avoided in uncontrolled proliferative retinopathy. Whereas, for people with kidney disease, physical activity can increase physical function and quality of life. Exercise can be performed partly during dialysis. The presence of microalbuminuria may necessitate restrictions on exercise [75].

## 9. Summary—Key Messages for the Person with Diabetes in Relation to Exercise and CGM

Measurement of the glucose level using a CGM system provides opportunities to re-flect on the effects of physical activity in patients with T1D and T2D. Thus, CGM presents a powerful tool for avoiding hyper- or hypoglycemia prior to, during, and after exercising. Moreover, it supports the decision-making of clinicians considering the patient’s indivi-dualized physical activity and its appropriate adjustment. Considering these CGM cha-racteristics and the advantages these systems allow, CGM’s role in analyzing physical activity’s effect on glucose levels will most certainly further increase in the future.

## 10. Conclusions

Physical activity is recommended in clinical practice guidelines as a preventive and therapeutic measure for the management of diabetes. This recommendation is supported by several studies. However, physical activity is challenging for patients with T1D and T2D, as they are at risk of triggering a blood sugar derailment. To avoid hypoglycemia during physical activity, people with diabetes need to adjust carbohydrate intake and insulin administration and make decisions based on real-time glucose levels. In this context, CGM systems have proven to be reliable in detecting glycemic fluctuations during exercise more quickly, as well as preventing the risk of hypoglycemia and treating hypoglycemic episodes immediately. Furthermore, CGM has proven to be helpful in monitoring TIR by the patient and the physician.

We conclude CGM systems are beneficial for people with diabetes and should be considered an important tool to assist people with diabetes during physical activity.

## Figures and Tables

**Table 1:** Additional carbohydrate intake due to physical activity for insulin-dependent people with diabetes according to ADA recommendations [52].

Blood Glucose before Physical Activity	Specifications for Additional Carbohydrate Intake
<90 mg/dL (<5.0 mmol/L)	Before training start, consume 15–30 g fast-acting carbohydrates depending on intensity (e.g., high intensity due to weight training) and duration of physical activity (<30 min), subsequently no additional carbohydrate intake required For prolonged activities of moderate intensity, additional carbohydrates as needed (0.5–1.0 g/kg body mass per hour of training), keep in mind control measurement of glucose
90–150 mg/dL (5.0–8.3 mmol/L)	Carbohydrate uptake at the beginning of most physical activities (approximately 0.5–1.0 g/kg body mass per hour of exercise), consider type of exercise and amount of active insulin
150–250 mg/dL (8.3–13.9 mmol/L)	Start training and delay carbohydrate intake until blood glucose level is at <150 mg/dL (<8.3 mmol/L)
250–350 mg/dL (13.9–19.4 mmol/L)	Ketone test, do not perform a training session when moderate to large amounts of ketones are present Start physical activity at light to moderate intensity, postpone intense physical activity until glucose levels <250 mg/dL, as intense physical activity may increase risk for hyperglycemia
≥350 mg/dL (≥19.4 mmol/L)	Ketone test, do not perform a training session when moderate to large amounts of ketones are present If ketones are negative (or only trace), consider conservative insulin correction (e.g., 50% correction) before starting the training session, depending on active insulin status Start light to moderate physical activity, avoid intense exercise until glucose level drop

Adapted to Zaharieva and Riddell [52].

**Table 2:** Adjustments of meal insulin bolus rates depending on training duration and intensity according to ADA recommendations [21].

	Training Duration	
	30 min	60 min
Intensity of physical activity		
Low (ca. 25% VO_2_max *)	Minus 25%	Minus 50%
Moderate (ca. 50% VO_2_max)	Minus 50%	Minus 75%
High (ca. 70–75% VO_2_max)	Minus 75%	Not specified
Intensive (>80% VO_2_max)	Reduction is not recommended	Not specified

* Oxygen uptake capacity (VO_2_max) is the maximum amount of oxygen that can be taken in during maximum physical exertion. VO_2_max is measured by breathing gas analysis during gradually increasing endurance exercise. Adapted to Colberg et al. [21].

## Data Availability

Not applicable since this is a narrative review.
